# Supplementary material for: Host gastric corpus microenvironment facilitates Ascaris suum larval hatching and infection in a murine model
Source: PLoS Negl Trop Dis. 2024 Feb 7;18(2):e0011930. doi: 10.1371/journal.pntd.0011930 (PMC10878500; doi:10.1371/journal.pntd.0011930)
Supplement: S1 Fig — Wildtype mice were oral gavaged with 2,500 Ascaris eggs and euthanized at 12 hours, 24 hours or 4 days post infection. H&E staining were carried out on sections of different intestine tissues. (PDF) [file pntd.0011930.s001.pdf]

Supplement Figure 1

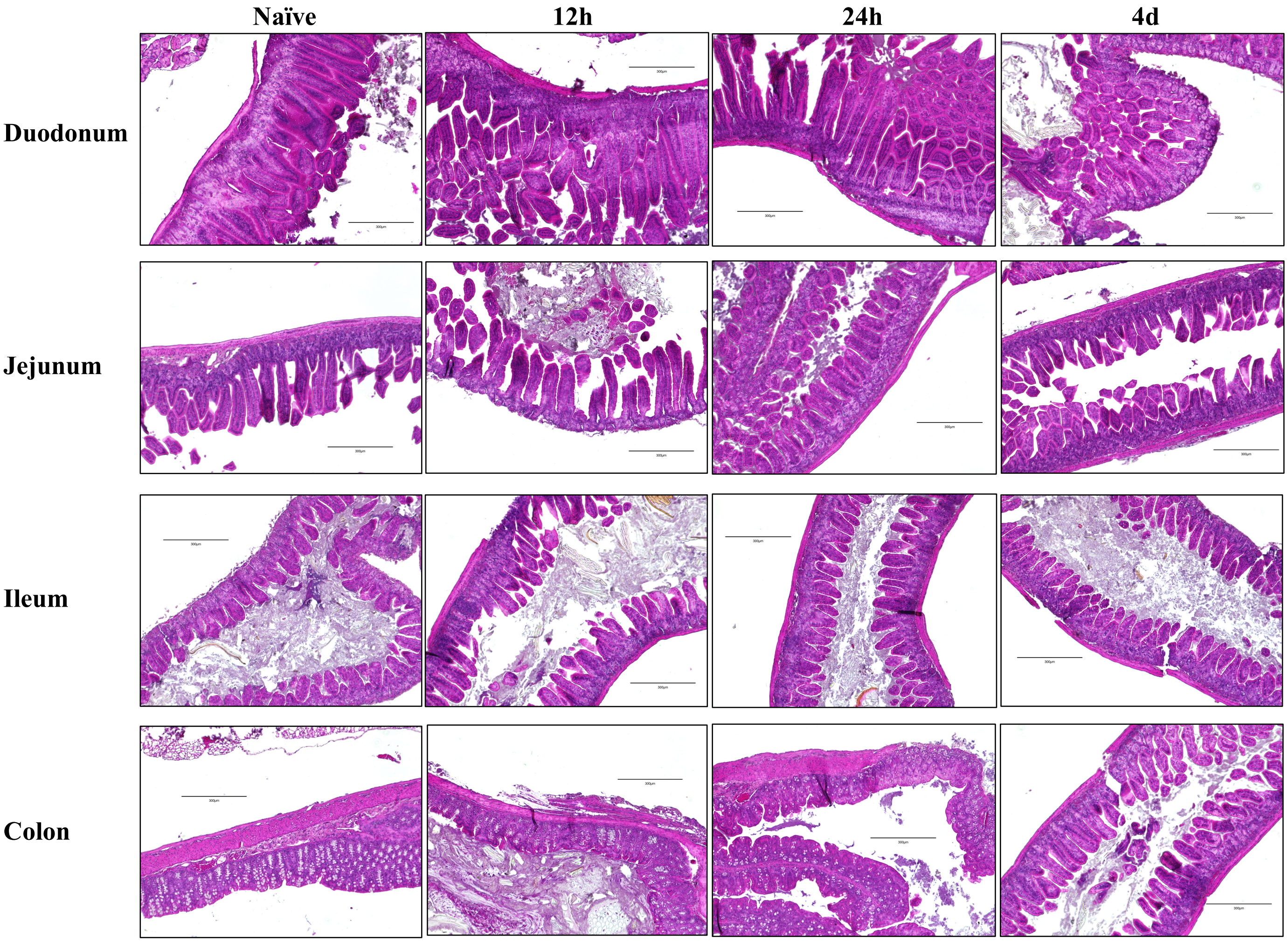

Supplemental Figure 1: Intestine of mice post *Ascaris* larva infection. Wildtype mice were oral gavaged with 2,500 *Ascaris* eggs and euthanized at 12 hours, 24 hours or 4 days post infection. H&E staining were carried out on sections of different intestine tissues.
