# Supplementary material for: Host gastric corpus microenvironment facilitates Ascaris suum larval hatching and infection in a murine model
Source: PLoS Negl Trop Dis. 2024 Feb 7;18(2):e0011930. doi: 10.1371/journal.pntd.0011930 (PMC10878500; doi:10.1371/journal.pntd.0011930)
Supplement: S2 Fig — Ascaris eggs are treated with 4 or 8 μg/ml of pepsin in pH = 2 overnight. Larvae hatched from the eggs were counted and hatch rate was calculated. (PDF) [file pntd.0011930.s002.pdf]

Supplement Figure 2

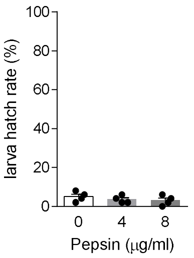

Supplemental Figure 2: Pepsin does not induce *Ascaris* larva hatching. *Ascaris* eggs are treated with 4 or 8 μg/ml of pepsin in pH=2 overnight. Larval hatch rate were then calculated.
