# Supplementary material for: Host gastric corpus microenvironment facilitates Ascaris suum larval hatching and infection in a murine model
Source: PLoS Negl Trop Dis. 2024 Feb 7;18(2):e0011930. doi: 10.1371/journal.pntd.0011930 (PMC10878500; doi:10.1371/journal.pntd.0011930)
Supplement: S3 Fig — Ascaris eggs were treated in pH = 2 for 30 minutes and then in different intestinal conditions overnight. Larval hatch rate was then calculated. (PDF) [file pntd.0011930.s003.pdf]

Supplement Figure 3

	Ctrl	Trypsin	Trypsin+Bile salt	Trypsin+Bile
Larval hatch rate %	0.315789%	0.27897%	0.506977%	0.217391%

Supplemental Figure 1: ***Ascaris*** **larva does not hatch in intestinal conditions.** Ascaris eggs are treated in pH=2 for 30 minutes and then in intestinal conditions overnight. Larval hatch rate were then calculated.
