# Supplementary material for: Host gastric corpus microenvironment facilitates Ascaris suum larval hatching and infection in a murine model
Source: PLoS Negl Trop Dis. 2024 Feb 7;18(2):e0011930. doi: 10.1371/journal.pntd.0011930 (PMC10878500; doi:10.1371/journal.pntd.0011930)
Supplement: Attachment — Submitted filename: Review comments.docx [file pntd.0011930.s004.docx]

**Please add page and line numbers in the manuscript.**
We have added page and line numbers in the manuscript

**There appears to be errors in citation in the Result section. For example, “Previously, we have reported … with allergic airway disease.”**
We have corrected the citation in the result section

**1.5 to 2 um” μm**
We have corrected the misspelt micrometer.

**“The role of AMCase in helminth immunity … during chronic infection.” References are needed.**

We have included the correct reference regarding AMCase and helminth immunity.
